# Preliminary Analysis of Placental DNA Methylation Profiles in Piglets with Extreme Birth Weight Variations

**DOI:** 10.3390/ani15152168

**Published:** 2025-07-23

**Authors:** Zhiyuan Zhang, Baohua Tan, Jiawei Su, Jiaming Xue, Liyao Xiao, Zicong Li, Linjun Hong, Gengyuan Cai, Ting Gu

**Affiliations:** 1 National Engineering Research Center for Breeding Swine Industry, College of Animal Science, South China Agricultural University, Guangzhou 510642, China; zhiyuanzhang@stu.scau.edu.cn (Z.Z.); tanbaohua@stu.scau.edu.cn (B.T.); 20232024019@stu.scau.edu.cn (J.S.); jiamingxue@stu.scau.edu.cn (J.X.); xlyao@stu.scau.edu.cn (L.X.); lizicong@scau.edu.cn (Z.L.); linjun.hong@scau.edu.cn (L.H.); cgy0415@scau.edu.cn (G.C.); 2 Guangdong Provincial Key Laboratory of Agri-Animal Genomics and Molecular Breeding, College of Animal Science, South China Agricultural University, Guangzhou 510642, China

**Keywords:** DNA methylation, pigs, placental development, transcriptome

## Abstract

Adequate birth weight is essential for animal survival and subsequent growth. However, the mechanism by which placental DNA methylation influences fetal growth remains incompletely understood. This study employed whole-genome bi-sulfite sequencing (WGBS) and RNA sequencing to analyze placental tissues from two weak piglets and two normal piglets born to the same sow. Transcriptome analysis identified 1989 differentially expressed genes (DEGs) enriched in blood/immune processes. Additionally, differentially methylated regions linked to DEG repression were enriched in extracellular matrix (ECM) receptors and angiogenesis pathways. To investigate the role of DNA methylation in gene regulation, porcine trophoblast cells (PTr2) were treated with either DMSO (control) or the DNA methylation inhibitor 5-Aza-2′-deoxycytidine (5-Aza). Real-time quantitative PCR (RT-qPCR) analysis demonstrated significant upregulation of *PACC1*, *SLC7A1*, and *PKP1* gene expression in the 5-Aza-treated group compared to controls (*p* < 0.05). Furthermore, methylation-specific PCR (MS-PCR) assays confirmed that the transcriptional activity of these genes is directly modulated by DNA methylation. These findings suggest that the dynamic regulation of DNA methylation in gene promoters may influence variations in placental morphology and birth weight in piglets, offering new insights into epigenetic regulation of fetal development, though larger studies are needed for validation.

## Simple Summary

A healthy birth weight is vital for newborn animals, but we still do not fully understand how epigenetic changes (DNA methylation) in the placenta influence fetal growth. In this study, we compared placentas from weak and normal piglets from the same mother and discovered nearly 2000 genes with different activity levels, many linked to blood and immune functions. Changes in DNA methylation were also tied to genes affecting placental structure and nutrient supply. In lab experiments, treating placental cells with a methylation-blocking compound activated key genes for nutrient transport and cell growth, proving that DNA methylation directly suppresses them. Our findings show that DNA methylation critically regulates placental function and birth weight. This research could help reduce low birth weight in livestock and deepen the understanding of how environmental factors affect fetal development in animals and humans. Further studies with larger groups are needed to confirm these results.

## 1. Introduction

Birth weight and overall fetal development are crucial neonatal health indicators with long-term effects on growth and disease susceptibility [1]. In livestock, particularly pigs, low birth weight and impaired development have been linked to poor neonatal outcomes, such as weak piglets that struggle with standing, have low body weight, and exhibit poor muscle development. These weak piglets face significant challenges in thriving at post-birth, raising concerns for animal welfare and agricultural productivity. Although the underlying mechanisms of these growth deficits are not fully understood, emerging evidence suggests that epigenetic modifications, particularly DNA methylation, play a vital role in regulating fetal development and placental function [2].

The placenta is a vital organ that facilitates nutrient and gas exchange between the mother and fetus, and beyond delivering essential nutrients, it regulates fetal development through endocrine and immune functions [3,4]. As gestation progresses, the fetal demand for nutrients increases rapidly, driving placenta remodeling to improve efficiency. This remodeling involves two key processes: (1) the formation of placental folds, which expand the maternal–fetal exchange surface area, and (2) the increase in capillary density and permeability [5]. Placental efficiency is critical for proper fetal growth, and impaired placental development is often linked to adverse birth outcomes, including low birth weight and retarded postnatal growth [6]. In particular, weak piglets typically exhibit underdeveloped muscle tissue and reduced body weight due to inadequate nutrient transfer during pregnancy and insufficient placental efficiency. Therefore, understanding the molecular mechanisms that regulate placental function, especially during periods of insufficient fetal growth, is crucial for improving neonatal outcomes. The porcine epitheliochorial placenta is particularly valuable for such investigations, as its unique non-invasive structure and prolonged gestation period allow detailed study of sustained nutrient transport mechanisms during late gestation, when fetal growth peaks.

DNA methylation is a key epigenetic modification central to regulating gene expression during placental development. It involves adding a methyl group from DNA to CPG dinucleotides, which can influence gene expression without altering the underlying genetic sequence [7]. In the placenta, DNA methylation regulates trophoblast invasion, placental growth, and the expression of genes involved in nutrient transport and fetal development [8]. Abnormal DNA methylation patterns in the placenta have been linked to various pregnancy complications and fetal growth disorders, including intrauterine growth restriction and low birth weight [9,10].

Recent studies have demonstrated that the DNA methylation landscape of the placenta can vary significantly between fetuses with different birth weights [11,12]. Weak piglets (characterized by low birth weight, poor viability, and high mortality) represent an ideal model for epigenetic functional studies due to their frequently appearing phenotypic variation within litters under standardized conditions [13]. We hypothesize that DNA methylation alterations in placental genes regulating nutrient transport and myogenesis are key factors leading to impaired fetal growth in weak piglets. Specifically, we propose the following: (1) differentially methylated regions (DMRs) in these functional pathways are directly associated with gene expression levels; (2) epigenetic dysregulation disrupts placental vascular development and nutrient permeability; (3) these molecular changes have a causal relationship with low birth weight and postnatal growth retardation. To test these hypotheses, we performed the following: (1) integrated analysis of weak placentas and normal placentas using WGBS and RNA-seq; (2) identification of DMRs associated with growth-related pathways; (3) verification that DNA methylation can regulate the expression of these candidate genes. Although previous studies have emphasized the role of DNA methylation in placental development and fetal growth, research directly comparing the DNA methylation profiles between weak and normal piglets remains limited.

This study integrated RNA-seq and WGBS to compare the placental DNA methylation profiles of weak and normal piglets, identifying specific methylation differences that may contribute to growth deficiency. Expression patterns and DNA methylation characteristics were analyzed between weak and normal piglets to reveal key molecular pathways and genes related to placental efficiency, nutrient transport, and muscle development. The integration of DNA methylation data with gene expression profiles may help improve our understanding of the epigenetic regulation of fetal growth. It improves livestock health and productivity by targeting epigenetic pathways during pregnancy.

## 2. Materials and Methods

### 2.1. Sample Collection

This study selected Large White pig gilts for research; placenta samples from weak and normal piglets were collected immediately after farrowing (within 30 min). Litters were randomly assigned to sampling groups. Biological replicates (*n* = 2/group) consisted of intentionally selected matched pairs from the same sow (two weak and two normal piglets per litter, representing the lower-weight (weak) and higher-weight (normal) phenotypes, respectively) to control for maternal effects while maintaining phenotypic contrast. These samples were excised from the maternal side of the placenta, 2 cm away from the umbilical cord insertion site, ensuring the absence of maternal decidua, corresponding to the chorioallantoic placenta [11]. All tissue samples were briefly rinsed with phosphate buffer solution, quickly sectioned into a 2 mL centrifuge tube, and stored at −80 °C.

### 2.2. H&E Staining and Immunohistochemistry

H&E staining: The histological examination was performed following standard H&E staining procedures as previously reported [14]. Tissue specimens embedded in paraffin were sectioned at 4 μm thickness and mounted on Superfrost Plus slides (Thermo Fisher Scientific, Waltham, MA, USA). After deparaffinization and rehydration, the sections were stained with hematoxylin for 15 min, followed by rinsing under running water. Subsequent counterstaining was performed using eosin, followed by dehydration through an ethanol series (70%, 95%, and 100%; 20 s each) and clearing in xylene (3 min). Processed sections were permanently mounted with Permount™ mounting medium (Thermo Fisher Scientific, Waltham, MA, USA). Microscopic evaluation was conducted using a Nikon ECLIPSE Ci microscope (Nikon Corporation, Tokyo, Japan).

IHC: Paraffin sections were deparaffinized using an eco-friendly solution and ethanol, then washed. After antigen retrieval, endogenous peroxidase was blocked with 3% H_2_O_2_ for 25 min, followed by blocking with 3% BSA for 30 min. Primary antibodies (DNMT3A or DNMT3B, MCE, 1:200, Monmouth Junction, NJ, USA) were applied and incubated overnight at 4 °C. A secondary antibody (goat anti-rabbit IgG, 1:300, Servicebio, Wuhan, China) was then added and incubated at room temperature for 50 min. After DAB staining, sections were counterstained with hematoxylin for 3 min, dehydrated in alcohol and xylene, and mounted for microscopy. Positive signals appeared brownish-yellow, and nuclei were blue.

### 2.3. Real-Time Quantitative PCR

Total RNA was extracted using Trizol reagent (15596018, Gibco, Grand Island, NY, USA) following the manufacturer’s instructions.

RNA extraction from placental tissue: (1) Tissue processing: Approximately 30 mg of tissue was weighed, ground in liquid nitrogen, then lysed with 1 mL of Trizol and transferred to a 1.5 mL nuclease-free centrifuge tube. (2) Lysis and phase separation: 200 μL of chloroform was added, mixed vigorously by vortexing, incubated for 3 min at room temperature, and centrifuged at 12,000× *g* for 15 min at 4 °C. The aqueous phase was transferred to a new tube. (3) RNA precipitation: An equal volume of isopropanol was added to the supernatant, mixed by inversion, and incubated on ice for 10 min. Centrifuged at 12,000× *g* for 15 min at 4 °C. The supernatant was discarded, retaining the white RNA pellet. (4) RNA washing: The pellet was washed twice with 1 mL of 75% ethanol (resuspended by pipetting), followed by centrifugation at 7500× *g* for 5 min at 4 °C. (5) RNA dissolution: The pellet was dissolved in 20 μL of DEPC-treated water by repeated pipetting, and concentration was measured.

RNA extraction from PTr2 cells: (1) Cell washing: Culture medium was removed, and cells in 6-well plates were washed twice with 1 mL of DPBS per well. (2) Cell lysis: 1 mL of Trizol was added per well, and adherent cells were detached by pipetting. Lysates were transferred to 1.5 mL of nuclease-free tubes, vortexed, and incubated for 10 min at room temperature. (3) Subsequent steps followed the tissue RNA extraction protocol (steps 2–5 above).

cDNA synthesis and RT-qPCR Analysis: The extracted RNA was reverse transcribed into cDNA using the Evo M-MLV Reverse Transcription Kit according to the manufacturer’s protocol (Accurate Biotechnology, Guangzhou, China). The resulting cDNA products were subsequently used as templates for quantitative RT-qPCR analysis to determine relative gene expression levels. RT-qPCR was conducted on an ABI QuantStudio 5 Flex system (Thermo Fisher Scientific, Waltham, MA, USA) using PowerUp™ SYBR™ Green Master Mix (Thermo Fisher Scientific, Waltham, MA, USA). Each time point was analyzed with three biological replicates, and each replicate included three technical replicates. Relative gene expression levels were calculated using the Ct (2^−ΔΔCt^) method. Primer sequences are listed in Supplementary Table S6.

### 2.4. RNA-Seq and Data Analysis

RNA quality assessment and sequencing: RNA concentration and integrity were measured with the RNA Nano 6000 Assay Kit (Agilent Technologies, Santa Clara, CA, USA) on a Bioanalyzer 2100 system. cDNA libraries were prepared with the NEBNext^®^ Ultra™ RNA Library Prep Kit (E7775, NEB, Ipswich, MA, USA) and subjected to 150 bp paired-end sequencing on an Illumina NovaSeq 6000 instrument (Novogene, Beijing, China).

Data processing pipeline: Initial quality control involved adapter trimming, poly-N sequence removal, and low-quality read filtration using fastp (version 0.23.1) [15]. Processed reads were then mapped to the Sus scrofa reference genome (Ensembl release 11.1.94) with Hisat2 (version 2.2.1) [16]. Gene-level counts were generated by featureCounts and subsequently normalized to TPM values through custom Perl/Python scripts (version 2.0.1) [17]. Differential gene expression analysis was performed using the DESeq2 R package (v1.34.0) [18], with genes meeting the criteria of adjusted *p*-value < 0.05 and absolute log2 fold change (|log_2_FC|) ≥ 1 defined as differentially expressed. 

### 2.5. Genome Bisulfite Sequencing and Data Analysis

Genomic DNA (100 ng), spiked with 0.5 ng of lambda DNA, was sonicated to a 200–300 bp fragment using a Covaris S220 (Covaris, Woburn, MA, USA). The DNA fragments were treated with bisulfite using the Scale Methyl-DNA Lib Prep Kit (RK20220, Abclonal, Wuhan, China) for Illumina, followed by library construction at Novogene Corporation (Novogene, Beijing, China). The samples were sequenced on an Illumina Novaseq platform (Illumina, San Diego, CA, USA) using 150 bp paired-end sequencing. Raw sequences in FASTQ format were pre-processed using fastp [15]. Clean reads were obtained by trimming adapter sequences, poly-N, and low-quality reads. Bisulfite-treated reads were aligned to the reference genome using Bismark software (version 0.19.0) with default parameters [19]. Duplicate reads aligning to the same regions of the genome were removed. DMRs were identified, and PCA was performed using the MethyKit R package (version 1.27.0) [20]. DMRs were annotated, and nearby genes were identified with the ChIPseeker R package (version 1.39.0) [21]. IGV software (version 2.10) was used to visualize the DNA methylation and expression patterns in selected regions.

To elucidate the biological functions of DMRs, GO and KEGG pathway analyses were conducted using the clusterProfiler R package (https://github.com/junjunlab/GseaVis accessed on January 2025) [22]. The GO analysis systematically examined three functional categories: biological process, molecular function, and cellular component. Pathway enrichment significance was determined at a false discovery rate (FDR) threshold of <0.05, with visualization of results through dot plots and bar graphs. Genes showing FPKM ≥ 0.5 in at least one sample were considered expressed. Alternative splicing (AS) events and differentially alternative splicing (DAS) events were identified using rMATS software (v4.1.2) [23] with updated genome annotation files (GTF). Software usage period: 2024–2025 (Last executed: 10 February 2025).

### 2.6. Cell Culture

PTr2 cells, derived from the filamentous embryos of pigs on day 12 of pregnancy, were generously provided by the team of Academician Yin Yulong at the Institute of Subtropical Agriculture, Chinese Academy of Sciences. PTr2 cells were maintained in DMEM/F12 medium (supplemented with 10% fetal bovine serum and 0.1% insulin, Gibco, Grand Island, NY, USA) at 37 °C in a 5% CO_2_ humidified atmosphere. When reaching 80% confluence, cells were passaged at a 1:3 ratio using 0.25% trypsin digestion for subsequent experiments. For DNA methylation inhibition, PTr2 cells were treated with 5-Aza (Sigma-Aldrich, Saint Louis, MO, USA) for 5 days with daily medium changes.

### 2.7. Methylation-Specific PCR

(1) Genomic DNA from weak and normal placentas was extracted and bisulfite-treated using the EZ DNA Methylation-Direct Kit (Zymo Research, Irvine, CA, USA) according to the manufacturer’s instructions.
(2) Specific MS-PCR primers were designed using MethPrimer [24] and are listed in Supplementary Table S6.
(3) PCR products were analyzed by 3% agarose gel electrophoresis (150 V) with 6× loading buffer (1:5 ratio). Samples were loaded with a DNA marker and visualized after electrophoresis.
(4) Methylation status was determined as follows: Unmethylated: Only unmethylated bands visible; Fully methylated: Only methylated bands visible; Partially methylated: Both methylated and unmethylated bands present.
(5) Methylation level was quantified using grayscale analysis: Methylation level (%) = [Methylated band intensity/(Methylated + Unmethylated band intensities)] × 100.

### 2.8. Statistical Analysis

Statistical analysis was performed using Statistical Package for the Social Sciences software (version 20.0, IBM, Armonk, NY, USA). The independent two-sample Student’s *t*-test was applied in this study to assess: (1) differences in gene and protein expression between placental tissues of weak piglets and normal piglets; (2) variations in protein expression among different placental tissue types; and (3) 5-Aza-induced alterations in candidate gene expression in PTr2 cells. Statistical significance levels were indicated as: * *p* < 0.05, ** *p* < 0.01, and *** *p* < 0.001, while ‘ns’ denoted non-significant results.

## 3. Results

### 3.1. Morphology and Gene Expression Changes Between Placentas from Weak and Normal Piglets

**Figure 1:**
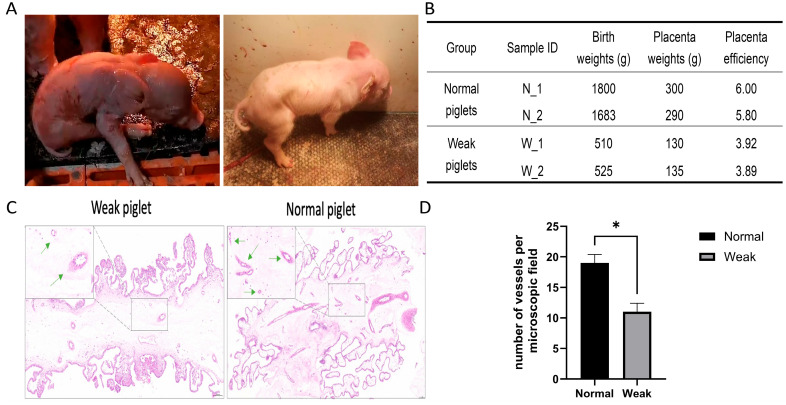
Morphological differences between placentas from weak and normal piglets. (**A**) Characteristics of weak and normal piglets. (**B**) Photographs of weak and normal piglets. (**C**) H&E staining exhibiting placental morphological changes at 5× magnification, with further detail at 20× magnification (upper-left panels). Green arrows indicate the placental vessels. (**D**) Histograms indicating placental vascular density in each group. * *p* < 0.05.

This study collected placental samples from both weak and normal piglets for sequencing. At sampling, normal piglets were selected from the same litter, characterized by vigor, good health at birth, and a weight of 1.6 kg or more. Simultaneously, piglets with significantly smaller size and lower weight than their littermates were considered weak, exhibiting unsteady limbs, trembling, and an absence of feeding behavior or inability to feed. Normal piglets exhibited higher birth and placental weights and efficiency than weak piglets (Figure 1A,B). Placental efficiency was calculated as fetal weight divided by placental weight. The morphological characteristics of porcine placental tissue were microscopically examined using hematoxylin and eosin (H&E) staining (Figure 1C). Placentas from normal piglets displayed a higher presence of nucleated red blood cells within the vessels and the intervillous space than those from weak placentas (Figure 1D).

**Figure 2:**
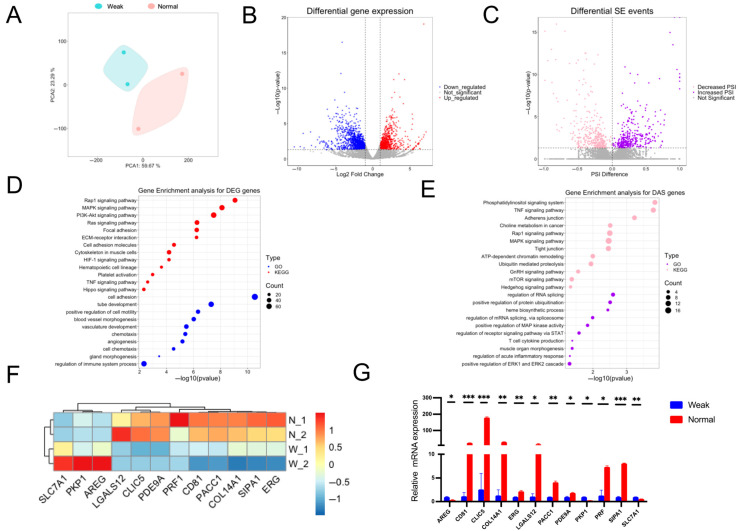
Dynamic gene expression changes between placentas from weak and normal piglets. (**A**) PCA results of gene expression data for each sample. (**B**) Volcano plot of DEGs. (**C**) Volcano plot of differentially alternatively spliced events (DASs). (**D**,**E**) GO and KEGG pathway enrichment analyses of DEGs (**D**) and DASs (**E**). (**F**) Heatmap exhibiting expression levels of selected DEGs. (**G**) The RT-qPCR validation of selected DEGs, * *p* < 0.05, ** *p* < 0.01, *** *p* < 0.001.

A total of 240,074,044 clean reads were generated from four porcine placenta samples derived from both weak and normal piglets (Supplementary Table S1). Principal component analysis (PCA) plots demonstrated that the biological replicates clustered closely together, indicating high consistency (Figure 2A). A total of 1989 DEGs were identified between the two placenta-type samples (Supplementary Table S2). Additionally, 711 differential exon skipping events (SE-DAS) were detected in 608 differentially spliced genes (DASGs) (Supplementary Table S2). Gene ontology (GO) and Kyoto encyclopedia of genes and genomes (KEGG) pathway analyses were conducted to investigate the function of DEGs and DASGs. GO analysis of DEGs revealed significant enrichment in processes related to blood development and immune system functions (Figure 2D) (Supplementary Table S3). KEGG pathway analysis identified the enrichment in ECM-receptor interaction, Hippo signaling, PI3K/Akt, and HIF-1 signaling pathways (Figure 2D) (Supplementary Table S3). For DASGs, GO analysis indicated enrichment in processes related to mRNA splicing and protein ubiquitination (Supplementary Table S3). KEGG enrichment analysis identified significant pathways such as MAPK signaling, Rap1 signaling, and TNF signaling (Figure 2E) (Supplementary Table S3). Finally, several DEGs were randomly selected, and RT-qPCR confirmed that their relative expression levels were closely correlated with those derived from transcriptome sequencing (Figure 2F,G). These findings emphasize the dynamic changes in gene expression and alternative splicing (AS) between the placentas of weak and normal piglets.

### 3.2. Characteristics of DNA Methylome Between Placentas from Weak and Normal Piglets

**Figure 3:**
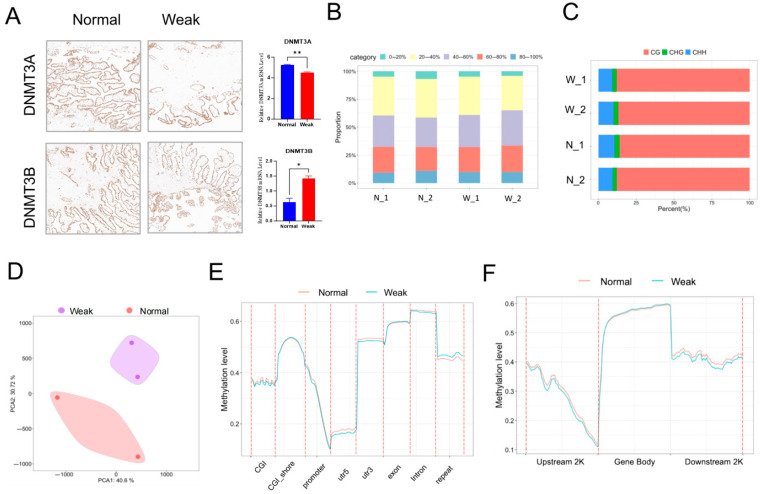
Characteristics of DNA methylome between placentas from weak and normal piglets. (**A**) IHC staining results for DNMT3A and DNMT3B, * *p* < 0.05, ** *p* < 0.01. (**B**) Histograms depicting the CpG methylation level of each sample. (**C**) Distribution of CpGs across different methylation levels in placenta tissues. (**D**) PCA of DNA methylation across each sample. (**E**,**F**) Average CpG methylation levels across different genomic features in weak and normal placentas.

Immunohistochemistry (IHC) experiments were performed to investigate the role of DNA methylation in placental development. The results revealed differential expression of the DNA methyltransferases 3A (DNMT3A) and DNMT3B between weak and normal placentas (Figure 3A). The DNA methylome characteristics of placentas from weak and normal piglets were subsequently examined. WGBS generated 1.21 billion paired-end reads (150 bp × 2), covering 328 Gb of sequence data (Supplementary Table S1). Genome-wide CpG methylation levels ranged from 48.8% to 50.7% across the different placenta samples, with most CpG sites exhibiting heavy methylation (40–80%) (Figure 3B). Most methylated cytosine sites were found in the CpG sequence context (approximately 80%), leading to a focus on methylated CpG sites (CpGs) for subsequent analysis (Figure 3C). PCA clearly separated replicates from different samples, with two biological replicates from each stage clustering together (Figure 3D). Unmethylated CpGs were primarily located in CpG islands (CGIs), gene promoters, and 5′ untranslated regions (UTRs), whereas CpGs in gene bodies, 3′ UTRs, CGI shelves, and CGI shores were highly methylated. Additionally, slightly higher methylation levels were observed in the UTR regions of normal placentas compared to weak placentas (Figure 3E,F). These findings reveal the distribution of methylated cytosine sites and CpG methylation levels across different genomic features.

### 3.3. Differences in DNA Methylation Between Placentas from Weak and Normal Piglets

**Figure 4:**
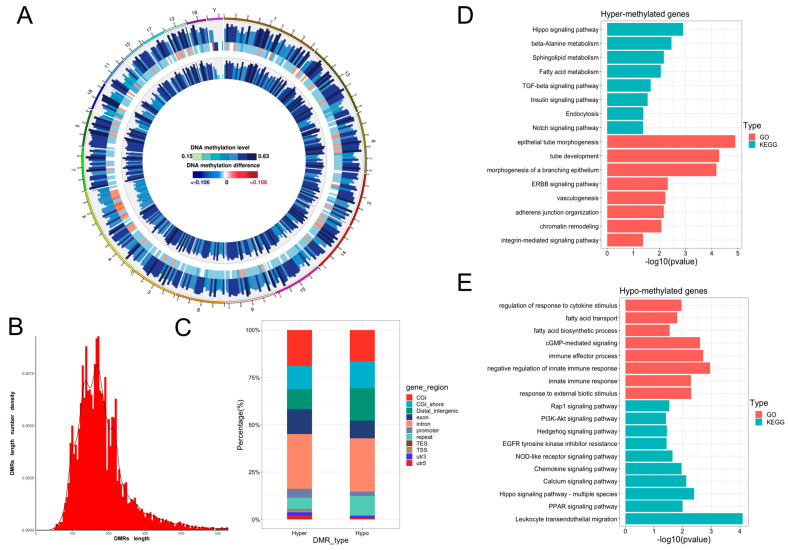
Regulation of DNA methylation in gene transcription in placentas from weak and normal piglets. (**A**) Circos plot depicting the distribution of DMRs across all chromosomes. (**B**) Length distribution of DMRs. (**C**) Genomic features distribution of DMRs. (**D**,**E**) GO and KEGG enrichment analyses of genes with DMRs in promoter regions.

DMRs were identified, and their functional significance in placental development was evaluated to investigate the dynamic changes and potential roles of DNA methylation (Supplementary Table S4). A comparison of placenta tissues from normal and weak piglets revealed 25,111 DMRs across the entire genome. A circos plot visualized the genome distribution of these DMRs, indicating their presence on all chromosomes without any clear chromosomal preference (Figure 4A). Most of the DMRs ranged in length from 100 to 300 bp (Figure 4B). Among these, 9564 were hypermethylated DMRs (hyper-DMRs), and 15,545 were hypomethylated DMRs (hypo-DMRs) (Figure 4C). Approximately 5% of these DMRs were located in gene promoters, while the remaining were found in introns and distal intergenic regions (Figure 4C). KEGG and GO enrichment analyses revealed significant enrichment of genes with hyper-DMRs in their promoters in pathways such as the integrin-mediated signaling pathway and vasculogenesis (Figure 4D). Furthermore, genes with hypo-DMRs in the promoter region were significantly enriched with fatty acid transport, immune response, and PI3K-Akt signaling pathways (Figure 4E). These findings highlight the dynamic nature of DNA methylation and its potential role in shaping the characteristics of placentas from weak and normal piglets.

### 3.4. Regulation of DNA Methylation in Gene Transcription in Placentas from Weak and Normal Piglets

**Figure 5:**
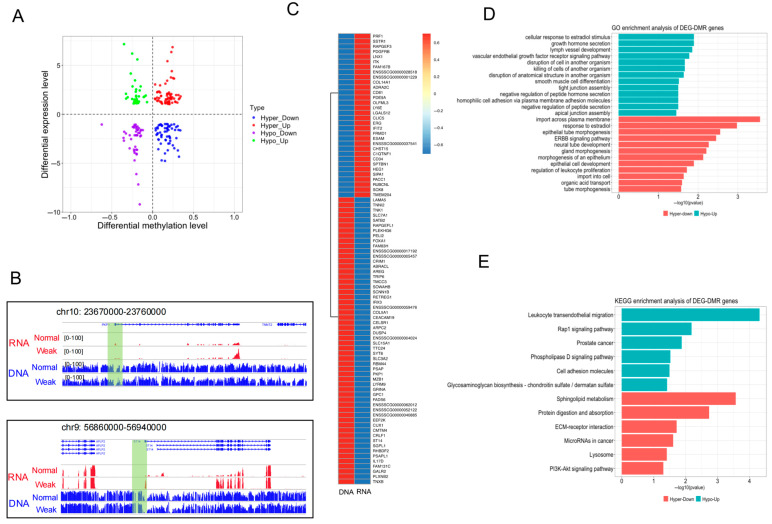
Validation of genes regulated by DNA methylation. (**A**) Four-quadrant plot displaying genes with significant changes in methylation and expression levels in the weak and normal piglets, with different types of genes represented in different colors. (**B**) Visualization of WGBS and RNA-seq tracks for *PKP1* and *ST14* gene in weak and normal placentas, with promoter DMRs highlighted in green. (**C**) Heatmap illustrating the expression of levels of selected DE-DMGs. (**D**,**E**) GO and KEGG enrichment analysis of DM-DEGs.

Integration of DNA methylome and transcriptome data identified differentially expressed and methylated genes (DM-DEGs) between the two types of placentas (Supplementary Table S5). A total of 206 DM-DEGs containing at least one promoter-DMR were identified, including 68 upregulated DEGs with hyper-DMRs, 60 downregulated DEGs with hyper-DMRs, 43 upregulated DEGs with hypo-DMRs, and 35 downregulated DEGs with hypo-DMRs (Figure 5A). Differential DNA methylation levels and expression profiles of two placenta-related DM-DEGs, *PKP1* and *ST14*, are presented in Figure 5B. Since DNA methylation is a well-established epigenetic marker closely associated with transcriptional repression [25], particular attention was given to the 88 DM-DEGs whose promoter DNA methylation levels exhibited a negative correlation with their expression levels. Hierarchical clustering based on gene expression revealed distinct differences in DNA methylation and expression levels of these DM-DEGs (Figure 5C). Functional analysis of these DM-DEGs through GO and KEGG enrichment identified their involvement in key placental development, including ECM-receptor interaction, cell adhesion, tight junction assembly, and gland morphogenesis (Figure 5D,E) (Supplementary Table S5). These findings highlight essential candidate genes regulated by DNA methylation that contribute to placental development.

### 3.5. Validation of DNA Methylation-Regulated Genes

**Figure 6:**
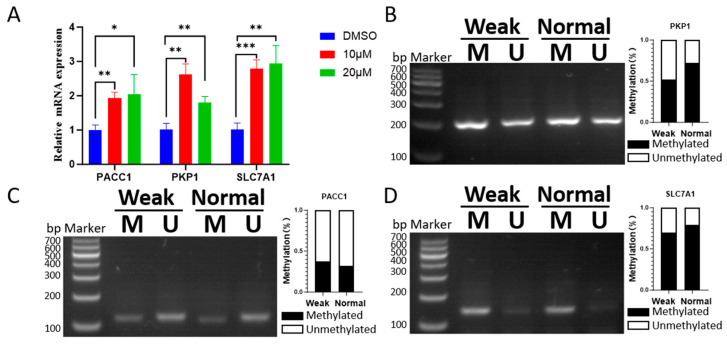
Relationship between DNA methylation and gene expression in placental development. (**A**) mRNA expression changes in selected DE-DMGs after 5-Aza-induced demethylation in PTr2 cells. * *p* < 0.05, ** *p* < 0.01, *** *p* < 0.001. (**B**–**D**) MSP analysis of the *PKP1*, *PACC1*, and *SLC7A1* promoter in placentas from weak and normal piglets. M: methylated alleles; U: unmethylated alleles.

To confirm the impact of DNA methylation on the DM-DEGs identified in this study, MS-PCR was performed to validate the differential methylation levels of *PKP1*, *PACC1*, and *SLC7A1*. PTr2 were treated with 5-Aza, an inhibitor of DNMTs, to evaluate the effects of DNA methylation on these genes. As anticipated, findings revealed that *PKP1*, *PACC1*, and *SLC7A1* exhibited upregulation following 5-Aza treatment (Figure 6A–D). Conclusively, this study successfully identified and validated several key genes regulated by DNA methylation that play a role in placental development.

## 4. Discussion

The present study investigated the microstructural differences, global-scale distributions of the DNA methylome profile, and corresponding transcriptome characteristics in the placentas of weak and normal piglets. DEGs were compared between the two placentas to identify the candidate genes involved in fetal development and placental function, revealing significant enrichment in processes such as blood vessel morphogenesis, cell adhesion, and ECM assembly. Suppression of placental angiogenesis has been reported to reduce blood flow to the placenta, leading to pregnancy complications and fetal growth failure [26]. As expected, a comparison of placental structures revealed a higher number of nucleated red blood cells in the normal placentas than in the weak placentas. The placenta is an immune barrier, protecting the fetus from invading pathogens [27]. Trophoblast cells secrete essential cytokines that play a critical role in maintaining normal gestation, and disruptions in this process can impair immune infiltration, leading to adverse pregnancy outcomes [28]. Gene functional enrichment analysis of the DEGs further revealed significant associations with vasculogenesis and immune-related processes, highlighting the critical role of proper vasculogenesis and immune response for both placental and fetal development. These results demonstrate that impaired placental angiogenesis coupled with dysregulated immune modulation collectively represent the key causative factors underlying neonatal piglet developmental disparities.

As has been identified as a key feature in placental diseases [29,30]. This study identified several genes exhibiting differential AS events between weak and normal piglet placentas. The DASGs were significantly enriched in mRNA splicing and protein ubiquitination processes. Notably, the pathways associated with DASGs differed significantly from those identified for DEGs, emphasizing the distinct molecular mechanisms underlying gene transcription and AS in the placenta, which warrant further investigation. A set of genes exhibiting a strong negative correlation with DNA methylation at their corresponding promoters was identified, highlighting the critical role of DNA methylation in regulating gene transcription during placental development. The findings revealed that DMRs are widespread in placental tissues, and genes annotated by DMRs are closely linked to placental development. The placental transfer of amino acids via amino acid transporters is essential for fetal growth [31]. These transporters work together to facilitate amino acid movements across the microvillous and basal plasma membranes of the placenta [32], with their expression and activity increasing throughout gestation [33]. Growing evidence indicates that DNA methylation can influence gene expression by modifying chromatin accessibility, ultimately leading to gene silencing. *SLC7A1*, a key gene in the cation transport system of the human placenta [34], was found to be regulated by DNA methylation and highly expressed in the placentas of weak piglets. This finding offers new insights into the function and regulation of *SLC7A1* during placental development.

This study has several limitations that warrant consideration. First, the minimal sample size (two piglets per group) may undermine statistical power and limit the generalizability of findings. The observed epigenetic patterns, while statistically significant, should be considered as preliminary data specific to extreme weight contrasts. Second, although numerous DMRs and genes were identified, the lack of comprehensive functional validation experiments, including gene knockdown and overexpression assays, hinders solid conclusions regarding underlying mechanisms. Therefore, future studies with larger sample sizes and more in-depth functional analyses are needed to validate and elucidate the regulatory pathways involved in fetal development.

## 5. Conclusions

Our integrated multi-omics analysis successfully achieved the study’s primary objective by identifying key epigenetic and transcriptional differences underlying extreme birth weight disparities in piglets (weak: 510–525 g vs. normal: 1683–1800 g, representing a >300% weight difference). The conserved regulatory mechanisms observed in porcine placental development offer direct translational value for understanding human fetal growth disorders, given the high evolutionary conservation of epigenetic pathways between species. To advance these findings, future studies should (1) conduct research with expanded sample sizes, (2) compare results with human placental epigenomes, and (3) investigate targeted interventions to improve fetal development.

## Data Availability

The datasets presented in this study are deposited in the NCBI Sequence Read Archive (SRA); the records can be accessed by accession numbers PRJNA1208693 and PRJNA1206694.

## Supplementary Materials

The following supporting information can be downloaded at https://www.mdpi.com/article/10.3390/ani15152168/s1, Table S1: Summary of the mapping of sequencing data; Table S2: The gene expression of identified DEGs; Table S3: GO and KEGG enrichment analysis of the DEGs; Table S4: Identification of DMRs; Table S5: Identification and functional enrichment analysis of DE-DMGs; Table S6: All primers used in this study.

## References

[1] Vilanova C.S., Hirakata V.N., de Souza Buriol V.C., Nunes M., Goldani M.Z., da Silva C.H. (2019). The relationship between the different low birth weight strata of newborns with infant mortality and the influence of the main health determinants in the extreme south of Brazil. Popul. Health Metr..

[2] Reik W., Dean W. (2001). DNA methylation and mammalian epigenetics. Electrophoresis.

[3] Koukoura O., Sifakis S., Spandidos D.A. (2012). DNA methylation in the human placenta and fetal growth (review). Mol. Med. Rep..

[4] Apicella C., Ruano C.S.M., Méhats C., Miralles F., Vaiman D. (2019). The Role of Epigenetics in Placental Development and the Etiology of Preeclampsia. Int. J. Mol. Sci..

[5] Han K., Ren R., Cao J., Zhao S., Yu M. (2019). Genome-Wide Identification of Histone Modifications Involved in Placental Development in Pigs. Front. Genet..

[6] Fowden A.L., Sferruzzi-Perri A.N., Coan P.M., Constancia M., Burton G.J. (2009). Placental efficiency and adaptation: Endocrine regulation. J. Physiol..

[7] Moore L.D., Le T., Fan G. (2013). DNA methylation and its basic function. Neuropsychopharmacology.

[8] Branco M.R., King M., Perez-Garcia V., Bogutz A.B., Caley M., Fineberg E., Lefebvre L., Cook S.J., Dean W., Hemberger M. (2016). Maternal DNA Methylation Regulates Early Trophoblast Development. Dev. Cell.

[9] Lee S., Kim Y.N., Im D., Cho S.H., Kim J., Kim J.H., Kim K. (2021). DNA Methylation and gene expression patterns are widely altered in fetal growth restriction and associated with FGR development. Anim. Cells Syst..

[10] Fantone S., Giannubilo S.R., Marzioni D., Tossetta G. (2021). HTRA family proteins in pregnancy outcome. Tissue Cell.

[11] Tan B., Xiao L., Wang Y., Zhou C., Huang H., Li Z., Hong L., Cai G., Wu Z., Gu T. (2024). Comprehensive Analysis of Placental DNA Methylation Changes and Fetal Birth Weight in Pigs. Int. J. Mol. Sci..

[12] Tan B., Zhou C., Zang X., Zhao X., Xiao L., Zeng J., Hong L., Wu Z., Gu T. (2023). Integrated Analysis of DNA Methylation and Gene Expression in Porcine Placental Development. Int. J. Mol. Sci..

[13] Zhang X. (2021). Analysis of the reasons for the formation and prevention of weak piglets in pig farms. Chin. Livest. Poult. Breed..

[14] Luo N., Cheng W., Zhou Y., Gu B., Zhao Z., Zhao Y. (2021). Screening Candidate Genes Regulating Placental Development from Trophoblast Transcriptome at Early Pregnancy in Dazu Black Goats (*Capra hircus*). Animals.

[15] Chen S., Zhou Y., Chen Y., Gu J. (2018). fastp: An ultra-fast all-in-one FASTQ preprocessor. Bioinformatics.

[16] Kim D., Langmead B., Salzberg S.L. (2015). HISAT: A fast spliced aligner with low memory requirements. Nat. Methods.

[17] Liao Y., Smyth G.K., Shi W. (2014). featureCounts: An efficient general purpose program for assigning sequence reads to genomic features. Bioinformatics.

[18] Love M.I., Huber W., Anders S. (2014). Moderated estimation of fold change and dispersion for RNA-seq data with DESeq2. Genome Biol..

[19] Krueger F., Andrews S.R. (2011). Bismark: A flexible aligner and methylation caller for Bisulfite-Seq applications. Bioinformatics.

[20] Akalin A., Kormaksson M., Li S., Garrett-Bakelman F.E., Figueroa M.E., Melnick A., Mason C.E. (2012). methylKit: A comprehensive R package for the analysis of genome-wide DNA methylation profiles. Genome Biol..

[21] Yu G., Wang L.-G., He Q.-Y. (2015). ChIPseeker: An R/Bioconductor package for ChIP peak annotation, comparison and visualization. Bioinformatics.

[22] Yu G., Wang L.-G., Han Y., He Q.-Y. (2012). clusterProfiler: An R package for comparing biological themes among gene clusters. Omics J. Integr. Biol..

[23] Shen S., Park J.W., Lu Z.X., Lin L., Henry M.D., Wu Y.N., Zhou Q., Xing Y. (2014). rMATS: Robust and flexible detection of differential alternative splicing from replicate RNA-Seq data. Proc. Natl. Acad. Sci. USA.

[24] Li L.C., Dahiya R. (2002). MethPrimer: Designing primers for methylation PCRs. Bioinformatics.

[25] Yang X., Gao L., Zhang S. (2017). Comparative pan-cancer DNA methylation analysis reveals cancer common and specific patterns. Brief. Bioinform..

[26] Huang Z., Huang S., Song T., Yin Y., Tan C. (2021). Placental Angiogenesis in Mammals: A Review of the Regulatory Effects of Signaling Pathways and Functional Nutrients. Adv. Nutr..

[27] Svensson-Arvelund J., Mehta R.B., Lindau R., Mirrasekhian E., Rodriguez-Martinez H., Berg G., Lash G.E., Jenmalm M.C., Ernerudh J. (2015). The human fetal placenta promotes tolerance against the semiallogeneic fetus by inducing regulatory T cells and homeostatic M2 macrophages. J. Immunol..

[28] Repnik U., Tilburgs T., Roelen D.L., van der Mast B.J., Kanhai H.H., Scherjon S., Claas F.H. (2008). Comparison of macrophage phenotype between decidua basalis and decidua parietalis by flow cytometry. Placenta.

[29] Ruano C.S.M., Apicella C., Jacques S., Gascoin G., Gaspar C., Miralles F., Méhats C., Vaiman D. (2021). Alternative splicing in normal and pathological human placentas is correlated to genetic variants. Hum. Genet..

[30] Gong S., Gaccioli F., Dopierala J., Sovio U., Cook E., Volders P.-J., Martens L., Kirk P.D.W., Richardson S., Smith G.C.S. (2021). The RNA landscape of the human placenta in health and disease. Nat. Commun..

[31] Simner C., Novakovic B., Lillycrop K.A., Bell C.G., Harvey N.C., Cooper C., Saffery R., Lewis R.M., Cleal J.K. (2017). DNA methylation of amino acid transporter genes in the human placenta. Placenta.

[32] Cleal J.K., Glazier J.D., Ntani G., Crozier S.R., Day P.E., Harvey N.C., Robinson S.M., Cooper C., Godfrey K.M., Hanson M.A. (2011). Facilitated transporters mediate net efflux of amino acids to the fetus across the basal membrane of the placental syncytiotrophoblast. J. Physiol..

[33] Loubière L.S., Vasilopoulou E., Bulmer J.N., Taylor P.M., Stieger B., Verrey F., McCabe C.J., Franklyn J.A., Kilby M.D., Chan S.Y. (2010). Expression of thyroid hormone transporters in the human placenta and changes associated with intrauterine growth restriction. Placenta.

[34] Shimada H., Powell T.L., Jansson T. (2024). Regulation of placental amino acid transport in health and disease. Acta Physiol..

